# Development of a novel antimicrobial-releasing glass ionomer cement functionalized with chlorhexidine hexametaphosphate nanoparticles

**DOI:** 10.1186/1477-3155-12-3

**Published:** 2014-01-23

**Authors:** Edward R Hook, Olivia J Owen, Candice A Bellis, James A Holder, Dominic J O’Sullivan, Michele E Barbour

**Affiliations:** 1 Oral Nanoscience, School of Oral and Dental Sciences, University of Bristol, Lower Maudlin St, Bristol, BS1 2LY, UK; 2 Kemdent, Associated Dental Products Ltd, Cricklade Road, Purton, Wiltshire, SN5 4HT, UK

**Keywords:** Nanoparticles, Nanobiomaterials, Antimicrobial, Dentistry

## Abstract

**Background:**

Glass ionomer cements (GICs) are a class of dental biomaterials. They have a wide range of uses including permanent restorations (fillings), cavity linings, fissure sealants and adhesives. One of the most common reasons for replacing a dental restoration is recurrent bacterial tooth decay around the margins of the biomaterial. Therefore, a dental biomaterial which creates a sustained antimicrobial environment around the restoration would be of considerable clinical benefit. In this manuscript, the formulation of a GIC containing novel antimicrobial nanoparticles composed of chlorhexidine hexametaphosphate at 1, 2, 5, 10 and 20% powder substitution by mass is reported. The aim is to create GICs which contain chlorhexidine-hexametaphosphate nanoparticles and characterize the nanoparticle size, morphology and charge and the release of chlorhexidine and fluoride, tensile strength and morphology of the GICs.

**Results:**

The GICs released chlorhexidine, which is a broad spectrum antimicrobial agent effective against a wide range of oral bacteria, over the duration of the experiment in a dose-dependent manner. This was not at the expense of other properties; fluoride release was not significantly affected by the substitution of antimicrobial nanoparticles in most formulations and internal structure appeared unaffected up to and including 10% substitution. Diametral tensile strength decreased numerically with substitutions of 10 and 20% nanoparticles but this difference was not statistically significant.

**Conclusion:**

A series of GICs functionalized with chlorhexidine-hexametaphosphate nanoparticles were created for the first time. These released chlorhexidine in a dose-dependent manner. These materials may find application in the development of a new generation of antimicrobial dental nanomaterials.

## Background

Glass ionomer cements (GICs) are a class of biomaterial in widespread use in modern dentistry [1]. They are used for a multitude of applications including for filling cavities caused by tooth decay or wear, as cavity liners, as fissure sealants, and as cements to form an adhesive bond between the tooth and a prosthetic dental restoration such as a crown or bridge. GICs have several favorable properties which make them suitable for these applications: They are tooth colored and available in a range of shades to allow matching to a patient’s natural dentition, they have a good biocompatibility profile, and they have an inherent adhesion to enamel and dentine and thus require minimal preparation of the tooth surface prior to application. In comparison to the other main clinical material used for direct tooth-coloured restorations, methacrylate resin-based silica-filled composites, GICs are less compromised by moisture contamination. This means that the clinician does not have to comply with such stringent requirements to thoroughly dry the tooth and surrounding area, which is a significant benefit over the less forgiving resin-based materials, although of course moisture control and careful preparation of the field is important with GICs and indeed all restorative materials. Aesthetically GICs are generally considered good, although the opacity and thus the "life-like" appearance are inferior to that of the resin-based materials. As well as for the applications described above, GICs also find application in Atraumatic Restorative Treatment (ART), whereby a dental filling is placed rapidly and without the use of drills or anesthetics [2]. This is particularly beneficial for pediatric and elderly patients as well as those with dental anxiety or learning difficulties.

GICs are acid–base cements composed of glass filler particles and polyacid molecules. The setting of the material is initiated by mixing with water, whereupon the polyacid molecules dissociate and cause dissolution of the glass, resulting in the release of ions which allow cross-linking of the polyacids. The transition from viscous liquid to rigid solid takes place typically over around 2 minutes, although the reaction does not reach completion until several days have elapsed. Even after setting the GICs can still participate in ion exchange with the oral fluids [3]. This has the outcome that GICs can release and absorb fluoride; the glasses in GICs contain calcium fluoride which leaches soluble fluoride into the mouth during normal function. The fluoride in the glass can be replenished by exposure to fluoride-containing oral care products such as dentifrice and mouth rinse, thus creating a rechargeable fluoride "reservoir" and allowing for sustained release of fluoride in the vicinity of a GIC restoration. The intention, when GICs were first developed, was that this fluoride release would protect the surrounding tooth tissue from further decay. While there is no doubt that fluoride in drinking water and oral care products serves to protect the teeth and improve oral health at an individual and community level [4], clinical data is not supportive of an anti-caries effect of GICs [5], and a recent Cochrane Database Systematic Review could find no evidence to support any beneficial result of fluoride-releasing restorative materials [6].

A GIC which offers a genuinely antimicrobial and antibiofilm efficacy would be of considerable clinical benefit [7]. Such a material could reduce recurrent decay in the vicinity of a restoration and could provide an antibacterial seal under other materials, protecting the pulp from bacterial ingress. It could be useful in ART, and as a fissure sealant, providing a protective seal over the occlusal surfaces of caries-vulnerable teeth. The fact that ions can readily travel in and out of the material offers the opportunity to dope the cement with other soluble antimicrobials. New advances in nanotechnology may provide the means to developing such a material [8].

In this manuscript the development of a GIC which contains novel antimicrobial nanoparticles composed of chlorhexidine hexametaphosphate (CHX-HMP nanoparticles) is reported. A recent publication describes surface functionalization of other materials using CHX-HMP nanoparticles prepared using a similar technique, and it was found that they acted as slow release devices for soluble chlorhexidine (CHX) [9] which is a potent antimicrobial agent in widespread use in medicine and dentistry. The CHX-HMP nanoparticles are formed by a precipitation reaction on mixing of aqueous CHX and HMP solutions with HMP in excess. The aim of this study was to establish whether it was possible to create viable GICs containing CHX-HMP nanoparticles and to investigate the properties of those GICs. The ultimate aim of this work is to create a GIC which exhibits a lasting antimicrobial effect *in vivo* without compromising other useful properties of the material.

## Results and discussion

### Overview

GIC specimens with substitutions of 1, 2, 5, 10 and 20% CHX-HMP nanoparticles for GIC powder were successfully created and compared with unmodified GICs (0% substitution). Those with 30% substitution of CHX-HMP nanoparticles were difficult to handle and the set material was crumbly so these were discarded without further analysis.

### Chlorhexidine release

**Figure 1:**
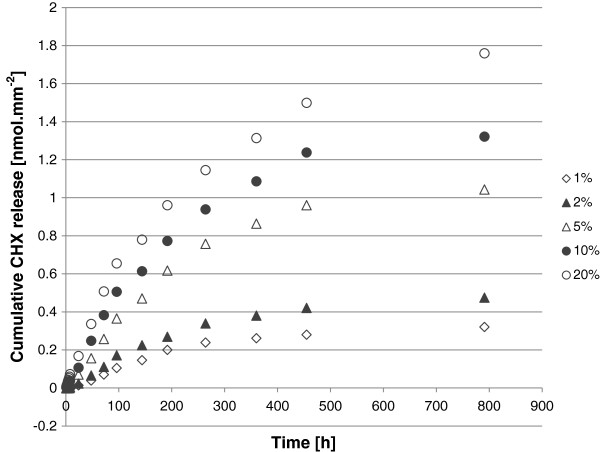
Cumulative CHX release from experimental GIC specimens with varying substitutions of CHX-HMP nanoparticles.

CHX release over 791 h (33 days) normalized to surface area and CHX-free controls is shown in Figure 1. CHX release persisted for the duration of the study with a rate of release which decreased with time. A dose–response was evident in that specimens with a higher substitution of CHX-NPs exhibited a larger CHX release, although the relationship was not directly proportional.

Cumulative CHX release at 1 and 24 h and 8, 15 and 33 days for the 6 specimen groups and the outcome of the statistical analyses are shown in Table 1. In this table, values for the 0% (unsubstituted) GICs are also shown; it can be seen that these readings were small compared to the actual CHX concentration readings, but these are included in the statistical analysis nevertheless so as not to manipulate the data unnecessarily and to allow a figure for comparison. The outcome was the same for each time point in that 0% and 1% were not statistically significantly different from one another, and 1% and 2% were not statistically significantly different from one another, but all other pairings were significantly different indicating a clear increase in chlorhexidine release correlated with an increase in nanoparticle substitution at all measured times.

**Table 1:** Cumulative CHX release for specimens with differing levels of nanoparticle substitution at each of 5 time points

	**Cumulative CHX release [nmol.mm**^**-2**^**] (standard deviation in parentheses)**				
**NP substitution [%]**	**1 h**	**24 h**	**8 days**	**15 days**	**33 days**
**0**	0.01 (0.003)^a^	0.01 (0.02)^a^	0.11 (0.06)^a^	0.12 (0.07)^a^	0.16 (0.08)^a^
**1**	0.16 (0.08)^a, b^	0.20 (0.09)^a, b^	0.48 (0.15)^a, b^	0.56 (0.16)^a, b^	0.65 (0.17)^a, b^
**2**	0.66 (0.18)^b^	0.70 (0.19)^b^	1.04 (0.21)^b^	1.17 (0.22)^b^	1.30 (0.24)^b^
**5**	1.30 (0.24)^c^	1.39 (0.25)^c^	2.03 (0.34)^c^	2.30 (0.36)^c^	2.51 (0.38)^c^
**10**	2.52 (0.38)^d^	2.65 (0.40)^d^	3.40 (0.43)^d^	3.73 (0.44)^d^	4.01 (0.45)^d^
**20**	4.02 (0.48)^e^	4.20 (0.50)^e^	5.09 (0.55)^e^	5.46 (0.59)^e^	5.94 (0.67)^e^

Within each time point, superscript letters indicate statistically homogeneous groups, so figures with different superscript letters are statistically significantly different to a 95% confidence level at that time.

Since CHX is efficacious against a wide range of bacteria and yeasts, this may confer antimicrobial and thus anti-caries properties on these nanofunctionalized dental filling materials. CHX disrupts the bacterial cell membrane [10] and results in the loss of intracellular components; this has the outcome that the evolution of bacterial resistance to CHX is considered unlikely [11].

Since CHX is an appealing option for the development of a dental cement which reduces the incidence of recurrent tooth decay, is it not surprising that there have been other attempts to incorporate CHX into GICs. CHX diacetate was added to a resin-modified GIC and this resulted in CHX release, but this was only sustained at significant levels for one week [12] and thus offered limited scope for lasting anti-caries effects. Incorporating CHX diacetate into a conventional (not resin-modified) GIC also yielded a CHX-releasing material, but again the CHX release was sustained for only around a week with all except the highest substitutions, and these high substitutions resulted in a deterioration of the mechanical properties of the material [13]. Dental composite resins supplemented with pulverized CHX diacetate also showed CHX release which reached a plateau after around 7 days [14]. The CHX release observed in the study reported here was more prolonged, and it is thought that this ie because the nanoparticlces themselves exhibit a gradual release of soluble CHX [9] rather than the already soluble and thus readily lost CHX in the studies described above.

Another report describes a longer-term effect of incorporating CHX – as ground CHX diacetate powder or as CHX digluconate solution – into GICs [15]. The CHX release per se was not measured but it was shown that an antimicrobial effect persisted for between 40 and 90 days. The peak of efficacy was the first 24 h for all GIC specimens, suggesting that most CHX may have been released during this initial period, and most specimens showed no antimicrobial behavior after 60–90 days. For some formulations, a limited deterioration in mechanical properties was observed. CHX digluconate solution has also been incorporated into GICs in combination with another antimicrobial agent, cetrimide, and this too had an antimicrobial effect on oral bacteria [16]. The authors indicate that this effect persisted for up to 180 days, but whether this was due to antimicrobial still leaching at the 180 day point, or that which had earlier leached and was still present in the agar plate, is not clear.

### Fluoride release

**Figure 2:**
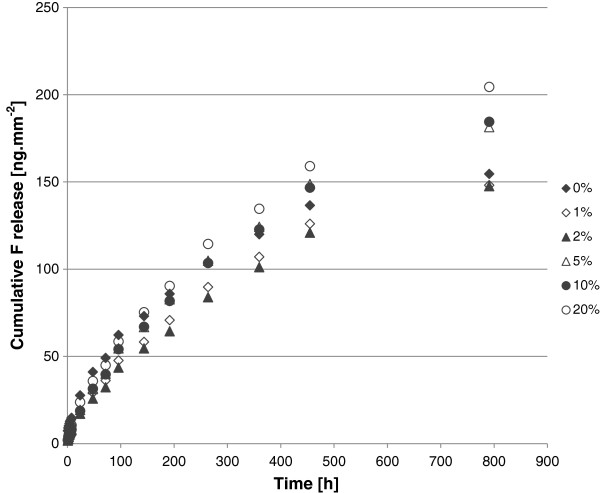
Cumulative fluoride release from experimental GIC specimens with varying substitutions of CHX-HMP nanoparticles.

Fluoride release over 791 h (33 days) normalized to surface area can be seen in Figure 2. All of the GIC specimens released fluoride continually over the duration of the experiment. The initial release rate was the most rapid and this gradually slowed over the experimental period, as has been observed for conventional GICs by other researchers [17].

Cumulative fluoride release at 1 and 24 h and 8, 15 and 33 days for the 6 specimen groups and the outcome of the statistical analysis are shown in Table 2. At 1 h, the unmodified GIC released significantly more fluoride than nanoparticle-substituted cements but there were no statistically significant differences between the different substitutions. At later times, in numerical terms fluoride release displayed a pattern of 20% > 5, 10% > 1, 2% with a complex relationship with 0%. However, there were few statistically significant differences; the only ones observed were at 24 h, 2% nanoparticle-substituted GICs released less fluoride than the 0% control, and at the longest time point, 33 days, the 20% nanoparticle-substituted GICs released more fluoride than the 2% nanoparticle-substituted GICs. It is not clear why the most highly substituted GIC released the most fluoride, especially as the greater the proportion of CHX-HMP nanoparticles, the smaller the total mass of fluoride-containing filler present. It is possible that the presence of the CHX-HMP nanoparticles alters the setting reaction and this renders fluoride more mobile in the cement lattice, but this is a hypothesis that has yet to be tested, and should be considered in the context that only the 2%-20% comparison showed significant differences. Although the impact of fluoride release from restorative materials is still a source of some controversy [1], it is not of concern in this study since the nanofunctionalized GICs showed similar fluoride release profiles to the unmodified cements. This is in contrast to an earlier report in which GICs supplemented with CHX digluconate solution exhibited a reduction in fluoride release [18].

**Table 2:** Cumulative fluoride release for specimens with differing levels of nanoparticle substitution at each of 5 time points

	**Cumulative fluoride release [ng.mm**^**-2**^**] (standard deviation in parentheses)**				
**NP substitution [%]**	**1 h**	**24 h**	**8 days**	**15 days**	**33 days**
**0**	7.38 (1.69)^a^	27.65 (0.85)^a^	85.82 (5.68)^a^	119.9 (11.0)^a^	154.5 (14.3)^a, b^
**1**	2.61 (1.11)^b^	18.59 (3.12)^a, b^	70.72 (8.40)^a^	106.9 (14.1)^a^	148.1 (16.6)^a^
**2**	1.68 (0.73)^b^	17.10 (2.33)^b^	64.30 (7.68)^a^	101.1 (12.2)^a^	147.5 (15.7)^a^
**5**	1.74 (0.70)^b^	19.31 (4.32)^a, b^	82.67 (7.49)^a^	124.2 (9.2)^a^	181.4 (14.0)^a, b^
**10**	1.83 (0.96)^b^	18.73 (9.50)^a, b^	81.73 (30.22)^a^	122.8 (39.3)^a^	184.4 (49.8)^a, b^
**20**	2.09 (0.34)^b^	23.74 (5.89)^a, b^	81.73 (13.15)^a^	134.6 (19.2)^a^	204.4 (23.4)^b^

Within each time point, superscript letters indicate statistically homogeneous groups, so figures with different superscript letters are statistically significantly different to a 95% confidence level at that time.

### Tensile strength

Diametral tensile strength of the 6 specimen groups are shown in Table 3. The ANOVA gave a p value of 0.054 indicating that, although there was a numerical trend towards lower tensile strength for 10 and 20% substitution cements, there was no statistically significant difference between these values and those of the other GICs. The fact that substitutions up to 5% appeared to have no significant deleterious effect on the tensile strength of the cements is encouraging.

**Table 3:** Diametral tensile strength of GIC specimens

**NP substitution [%]**	**Diametral tensile strength [MPa] (standard deviation in parentheses)**
0	14.1 (3.7)
1	14.3 (4.9)
2	15.7 (4.3)
5	15.5 (1.1)
10	11.5 (2.8)
20	9.4 (2.6)

The ANOVA gave p = 0.054 indicating that there were no statistically significantly differences between tensile strengths of the different GICs to a 95% confidence level.

### Nanoparticle characterization

**Figure 3:**
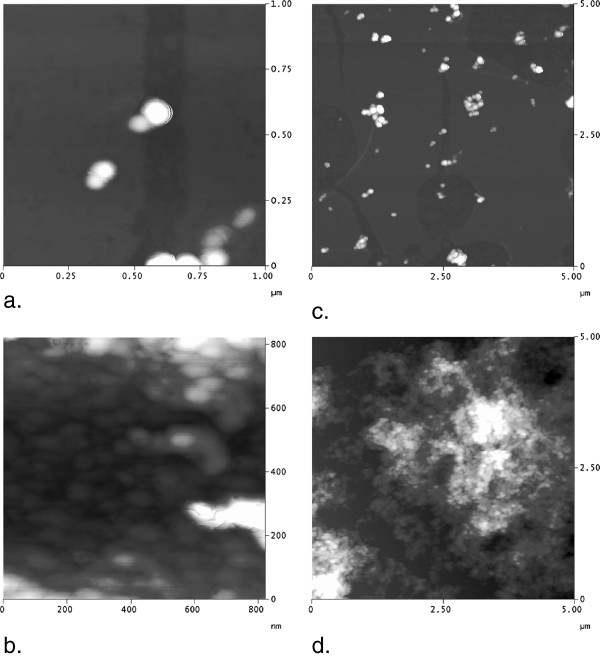
**Atomic force microscopy images showing CHX-HMP nanoparticles deposited on a glass coverslip. a**: 1 × 1 μm image with vertical scale 50 nm showing individual globular nanoparticles. **b**: 0.8 × 0.8 μm image with vertical scale 20 nm showing individual nanoparticles of similar shape and morphology as in **(a)** but in an aggregate. **c**: 5 × 5 μm image with vertical scale 100 nm showing individual nanoparticles and small aggregates. **d**: 5 × 5 μm image with vertical scale 500 nm showing nanoparticles in a large aggregate.

Dynamic light scattering (DLS) indicated that there were structures of mean diameter 196 nm, but it was observed that there was substantial polydispersity and the standard deviation was large (76 nm). The correlation functions observed during some DLS measurements suggested the presence of some much larger particles. This may be explained by observations made by atomic force microscopy (AFM) (Figure 3) which indicated that nanoparticles sometimes formed aggregates which could be as large as several micrometres. The individual nanoparticles which compose these aggregates were regularly shaped, globular and had typical diameters of 80–90 nm (Figure 3). This would not be observed in DLS since the signal is proportional to diameter to the 6th power, so the signal from these small nanoparticles would be masked by that from the larger aggregates.

Zeta potential measurements indicated that the nanoparticles had a mean surface charge of -55 mV (standard deviation 1.4 mV), indicating a net negative charge.

### Morphology and structure

**Figure 4:**
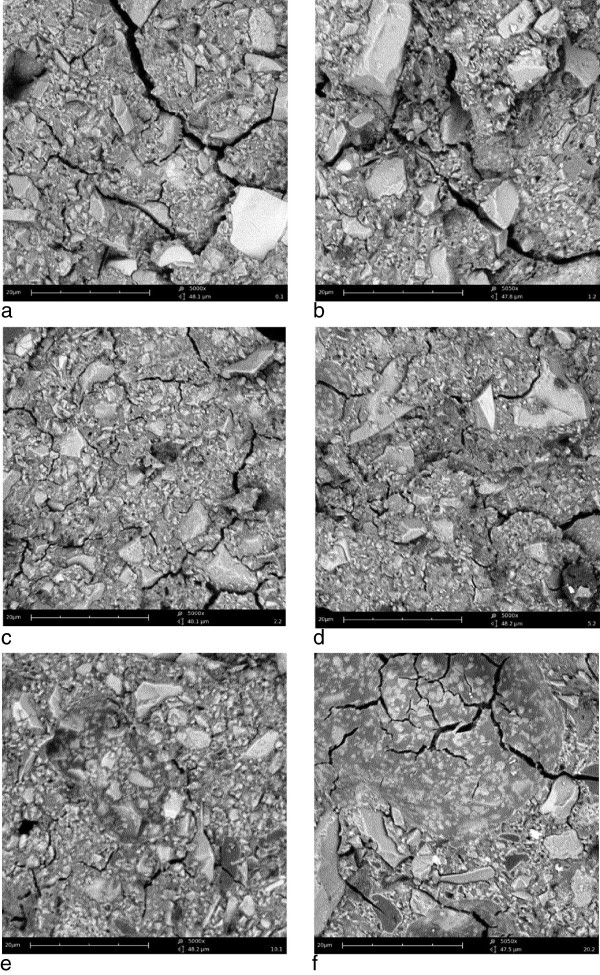
**Scanning electron micrographs showing fracture surfaces of GIC specimens. a**: unmodified GIC; **b**: 1% nanoparticles; **c**: 2% nanoparticles; **d**: 5% nanoparticles; **e**: 10% nanoparticles; **f**: 20% nanoparticles. The scale bar is 20 μm in each image.

Scanning electron micrographs of representative GIC specimens are shown in Figure 4. The appearances of the GIC specimens with different substitutions of nanoparticles were similar, with the glass filler particles and surrounding matrix clearly visible. Only the 20% nanoparticle substitution exhibited a slightly different appearance (Figure 4f), with textured areas depleted in glass particles and a smoother fracture plane which are suggestive of nanoparticle aggregates. It is possible that these could lead to a reduction in strength since they cannot be presumed to interact with the polyacid in the same way as the glass filler particles, and future studies will address this important question.

### Overview

By adding CHX-HMP nanoparticles to a commercial GIC it has proven possible to create a material which releases CHX for in a dose-dependent manner for longer than has been observed using some other approaches to CHX functionalization. The results of this study would suggest that substitutions of up to 20% nanoparticles for glass by mass using this approach may be suitable for further development as clinical materials. 10% and 20% substitutions showed a numerical reduction in strength but not a statistically significant one; subsequent follow-on studies will allow for further investigation of this observation. Higher substitutions, of 30% or more, are unlikely to find application without other changes to the cement as this resulted in a dry, crumbly cement with unacceptable handling properties. Options to use more dispersed nanoparticles, rather than the ground aggregates discussed here, are under investigation. Possibilities for CHX recharging and the microbiological impact of the CHX release are the subject of ongoing experiments, as well as larger studies to investigate the effect on different kinds of strength. Interestingly, it has recently been shown that resin-modified GICs can act as a short-term in vivo reservoir for topically applied CHX [19].

In this study, nanoparticles were substituted for powder by weight. An alternative approach is to substitute like-for-like by surface area, and this would account for the fact that the specific surface area of the nanoparticles is higher than that of the glass filler particles.

## Conclusions

Novel GICs have been created which contain antimicrobial CHX-HMP nanoparticles at a range of dopings. These GICs released soluble CHX over a period of at least 33 days, and the quantity of CHX released was dependent on the doping of nanoparticles in the cement. All cements released fluoride with a similar profile to the control, unmodified cement and moderate substitutions did not detrimentally affect the tensile strength of the material. These cements may find clinical application as dental biomaterials which prevent or reduce the incidence of secondary caries and protect the tooth and soft tissues from bacterial infection.

## Methods

### Synthesis and preparation of CHX-HMP nanoparticles

Aqueous stock solutions of chlorhexidine digluconate and sodium hexametaphosphate (Sigma Aldrich, Gillingham, UK) were mixed in deionized water such that the final concentration was 4 mM CHX and 5 mM HMP. The resulting colloidal suspension of CHX-HMP nanoparticles was mixed thoroughly and then centrifuged at 21000 g for 60 min. The supernatant was removed and discarded and the nanoparticle pellet dried for at least 48 h at 40°C. The pellet was then removed from the centrifuge tubes and ground to a fine white powder composed of nanoparticle aggregates using an agate mortar and pestle. Further details regarding the properties of CHX-HMP nanoparticles and their antimicrobial efficacy can be found elsewhere [9].

### Prototype nanofunctionalized glass ionomer cements

A commercially available GIC (Diamond Carve (TM), Kemdent, Purton, UK) was used as the starting material. This commercially available GIC comprises a powder, which consists of alumina-silica based glass filler particles containing calcium fluoride and other minor salts and freeze-dried poly (vinyl) phosphonic acid, and a liquid which contains polyacrylic and tartaric acids. It is mixed in a ratio of 1:4 liquid:powder by mass. Cylindrical GIC specimens with nominal dimensions of 6 mm diameter and 3 mm height were formed by mixing the GIC according to the manufacturers’ instructions and packing into Perspex molds coated with a thin layer of petroleum jelly to aid removal. The mixing was carried out by one individual (OJO) with extensive experience of GIC mixing and handling. The precise dimensions of each specimen were measured using calipers and recorded. The nanoparticle powder created by grinding the nanoparticle pellet and thus yielding compacted clusters of nanoparticles was used to substitute for the GIC powder at fractions of 0, 1, 2, 5, 10, 20 and 30% by mass. Ten specimens of each substitution were created giving a total of 70 specimens. They were removed from the mold within 60 minutes and placed in individual small, sealed plastic vessels that contained wet tissue paper not in direct contact with the specimen, to achieve an atmosphere of 100% humidity but prevent the specimen being in contact with liquid water which could result in dissolution during the critical early phases of setting. These were stored at 37°C for 7 days.

After this the specimens were divided into two sets of 5 specimens each. One set of each substitution was set aside for tensile strength and fracture surface morphology analysis and the other set was used to investigate the CHX and fluoride leaching from the cement.

For the investigations of CHX and fluoride release, each specimen was immersed in 1 mL artificial saliva in individually labeled vials at 37°C. The artificial saliva was composed of CaCl_2_ · 2H_2_O 0.103 gL^-1^, MgCl_2_ 0.019 gL^-1^, KH_2_PO_4_ 0.544 gL^-1^, C_8_H_18_N_2_O_4_S (HEPES buffer acidic form) 4.77 gL^-1^, KCl 2.24 gL^-1^, 1.80 mL 1 M HCl, KOH titrated to obtain a pH of 6.8. Specimens were periodically removed and placed in duplicate tubes containing fresh artificial saliva so that the artificial saliva the specimen had been incubated in could be sampled for CHX and fluoride concentrations. A pilot study was conducted to establish the saturation limit of fluoride concentration within the vessels to ensure that the sampling periods were selected appropriately and erroneous readings owing to saturation of the eluent by a fluoride salt were not obtained by leaving too large a gap between readings. Using the findings from this pilot study, the sampling occurred at hourly intervals during the first day, followed by intervals of 4 hours, then daily and then weekly. Controls containing only artificial saliva without a GIC specimen were sampled in the same way.

### Chlorhexidine measurements

CHX concentration in the artificial saliva was measured using ultraviolet spectrophotometry. The 1 mL artificial saliva was placed into a semi-micro cuvette transparent under ultraviolet wavelengths and absorption was measured at 255 nm using a spectrophotometer (Hitachi U1900, Tokyo, Japan). The reading was converted to CHX concentration with reference to calibration standards at 5–50 μmol.l^-1^. The concentration was converted to moles of CHX released per unit surface area of the GIC specimen with reference to the individual dimension measurements for each specimen and was normalized by subtracting the reading for 0% substitution to correct for any other molecules present in the system which absorbed at 255 nm such as the polyacrylic acid which is another component of the GIC.

### Fluoride measurements

Fluoride concentration in the artificial saliva was measured using an ion-selective electrode (9609BNWP, Thermo Fisher Scientific, Waltham, MA, USA) by mixing 0.5 mL artificial saliva with 0.5 mL TISAB solution (Thermo Fisher Scientific, Waltham, MA, USA). The data output was converted to mg/L fluoride ion with reference to calibration standards of 0.1, 0.5, 1, 2 and 5 mg/L F^-^, also diluted with equal quantities of TISAB.

### Tensile strength measurements

Indirect tensile strength (S_T_) was measured by applying a compressive diametric force to the curved sides of the cylindrical specimen (n = 5 per group) until fracture occurred, using a universal testing machine (LR5K, Lloyd Instruments, Ametek, FL, USA) recording the load at fracture (L) and using this with the specimen dimensions of height (h) and diameter (d) to calculate tensile strength according to the relationship:

$$\text{Sr}=\frac{2L}{\pi \text{dt}}$$

### Statistical analysis

Cumulative CHX and fluoride release at time points of 1 h, 24 h, 8 days, 15 days and 33 days, and indirect tensile strength, were compared using one-way ANOVAs with a Tukey honestly significant difference post-hoc test.

### Characterization of CHX-HMP nanoparticles

The size and zeta potential of the nanoparticles as prepared in colloidal suspension were measured using a Malvern Zetasizer (Malvern, UK). The size, morphology and aggregation of the nanoparticles were investigated when immobilized on glass coverslips. Coverslips were cleaned by ultrasonicating for 10 minutes in acetone followed by ultrasonicating for 10 minutes in ethanol then coated by dipping into a freshly prepared colloid of the nanoparticles for 30 seconds, then rinsing in running deionized water for 10 seconds, then allowing to dry in air. The nanoparticle-coated coverslips were imaged using AFM (Nanoscope IIIa, Digital Instruments, CA, USA).

### Morphology and structure

Specimens which had been tested for tensile strength were coated with a thin layer of gold-palladium (SC7620, Emitech, Taiwan) and examined using SEM (Phenom, Eindhoven, Netherlands). Images were obtained at nominal magnifications of 400, 1000 and 5000×.

## Abbreviations

CHX: Chlorhexidine; CHX-HMP: Chlorhexidine hexametaphosphate; GIC: Glass ionomer cement.

## Competing interests

The University of Bristol filed a patent application relating to the work presented in this manuscript in 2013.

## Authors’ contributions

ERH, OJO and CAB were the students who carried out the laboratory work and statistical analysis. JAH was the industry supervisor of the study, providing guidance and input on study design from the industrial perspective. DJOS was the clinical supervisor of the study, providing a clinician’s perspective on important material properties and helping to develop the study parameters and design. MEB was the principal, scientific supervisor of the study. She conceived the study, supervised the students in the laboratory, directed the analysis and wrote the manuscript. All authors read and approved the final draft of the manuscript.

## References

[B1] Sidhu SK Glass-ionomer cement restorative materials: a sticky subject? Aust Dent J 2011 56 1 23 30 10.1111/j.1834-7819.2010.01282.x 21564113

[B2] Holmgren CJ Roux D Domejean S Minimal intervention dentistry: part 5. Atraumatic restorative treatment (ART)–a minimum intervention and minimally invasive approach for the management of dental caries Br Dent J 2013 214 11 18 10.1038/sj.bdj.2012.1175 23306489

[B3] Billington RW Williams JA Pearson GJ Ion processes in glass ionomer cements J Dent 2006 34 544 555 10.1016/j.jdent.2005.09.008 16574301

[B4] Griffen AL Goepferd SJ Preventive oral health care for the infant, child, and adolescent Pediatr Clin North Am 1991 38 1209 1226 1886743 10.1016/s0031-3955(16)38195-0

[B5] Randall RC Wilson NH Glass-ionomer restoratives: a systematic review of a secondary caries treatment effect J Dent Res 1999 78 628 637 10.1177/00220345990780020101 10029460

[B6] Yengopal V Harneker SY Patel N Siegfried N Dental fillings for the treatment of caries in the primary dentition Cochrane Database Syst Rev 2009 CD004483 19370602 10.1002/14651858.CD004483.pub2

[B7] Wang Z Shen Y Haapasalo M Dental materials with antibiofilm properties Dent Mater 2013 30 e1 e16 24374001 10.1016/j.dental.2013.12.001

[B8] Melo MA Guedes SF Xu HH Rodrigues LK Nanotechnology-based restorative materials for dental caries management Trends Biotechnol 2013 31 459 467 10.1016/j.tibtech.2013.05.010 23810638 PMC3845439

[B9] Barbour ME Maddocks SE Wood NJ Collins AM Synthesis, characterization, and efficacy of antimicrobial chlorhexidine hexametaphosphate nanoparticles for applications in biomedical materials and consumer products Int J Nanomedicine 2013 8 3507 3519 24092973 10.2147/IJN.S50140 PMC3787925

[B10] Gilbert P Moore LE Cationic antiseptics: diversity of action under a common epithet J Appl Microbiol 2005 99 703 715 10.1111/j.1365-2672.2005.02664.x 16162221

[B11] Meyer B Cookson B Does microbial resistance or adaptation to biocides create a hazard in infection prevention and control? J Hosp Infect 2010 76 200 205 10.1016/j.jhin.2010.05.020 20638752

[B12] Sanders BJ Gregory RL Moore K Avery DR Antibacterial and physical properties of resin modified glass-ionomers combined with chlorhexidine J Oral Rehabil 2002 29 553 558 10.1046/j.1365-2842.2002.00876.x 12071924

[B13] Palmer G Jones FH Billington RW Pearson GJ Chlorhexidine release from an experimental glass ionomer cement Biomaterials 2004 25 5423 5431 10.1016/j.biomaterials.2003.12.051 15130727

[B14] Cheng L Weir MD Xu HH Kraigsley AM Lin NJ Lin-Gibson S Zhou X Antibacterial and physical properties of calcium-phosphate and calcium-fluoride nanocomposites with chlorhexidine Dent Mater 2012 28 573 583 10.1016/j.dental.2012.01.006 22317794 PMC3322264

[B15] Turkun LS Turkun M Ertugrul F Ates M Brugger S Long-term antibacterial effects and physical properties of a chlorhexidine-containing glass ionomer cement J Esthet Restor Dent 2008 20 29 44 discussion 45 10.1111/j.1708-8240.2008.00146.x 18237338

[B16] Korkmaz FM Tuzuner T Baygin O Buruk CK Durkan R Bagis B Antibacterial activity, surface roughness, flexural strength, and solubility of conventional luting cements containing chlorhexidine diacetate/cetrimide mixtures J Prosthet Dent 2013 110 107 115 10.1016/S0022-3913(13)60349-2 23929372

[B17] Yip HK Smales RJ Fluoride release from a polyacid-modified resin composite and 3 resin-modified glass-ionomer materials Quintessence Int 2000 31 261 266 11203934

[B18] Hoszek A Ericson D In vitro fluoride release and the antibacterial effect of glass ionomers containing chlorhexidine gluconate Oper Dent 2008 33 696 701 10.2341/08-20 19051864

[B19] Lim BS Cheng Y Lee SP Ahn SJ Chlorhexidine release from orthodontic adhesives after topical chlorhexidine treatment Eur J Oral Sci 2013 121 211 217 10.1111/eos.12033 23659245

